# Acceptability, effectiveness, and cost-effectiveness of internet-based exposure treatment for irritable bowel syndrome in a clinical sample: a randomized controlled trial

**DOI:** 10.1186/1471-230X-11-110

**Published:** 2011-10-12

**Authors:** Brjánn Ljótsson, Gerhard Andersson, Erik Andersson, Erik Hedman, Perjohan Lindfors, Sergej Andréewitch, Christian Rück, Nils Lindefors

**Affiliations:** 1 Department of Clinical Neuroscience, Division of Psychiatry, Karolinska Institutet, Stockholm, Sweden; 2 Department of Behavioural Sciences and Learning; Swedish Institute for Disability Research, Linköping University, Linköping, Sweden; 3 Department of Gastroenterology, Sabbatsberg hospital, Stockholm, Sweden

## Abstract

**Background:**

Internet-based cognitive behavior therapy (ICBT) has shown promising effects in the treatment of irritable bowel syndrome (IBS). However, to date no study has used a design where participants have been sampled solely from a clinical population. We aimed to investigate the acceptability, effectiveness, and cost-effectiveness of ICBT for IBS using a consecutively recruited sample from a gastroenterological clinic.

**Methods:**

Sixty-one patients were randomized to 10 weeks of ICBT (n = 30) or a waiting list control (n = 31). The ICBT was guided by an online therapist and emphasized acceptance of symptoms through exposure and mindfulness training. Severity of IBS symptoms was measured with the Gastrointestinal symptom rating scale - IBS version (GSRS-IBS). Patients in both groups were assessed at pre- and post-treatment while only the ICBT group was assessed 12 months after treatment completion. Health economic data were also gathered at all assessment points and analyzed using bootstrap sampling.

**Results:**

Fifty of 61 patients (82%) completed the post-treatment assessment and 20 of 30 patients (67%) in the ICBT group were assessed at 12-month follow-up. The ICBT group demonstrated significantly (*p *< .001) larger improvements on the IBS-related outcome scales than the waiting list group. The between group effect size on GSRS-IBS was Cohen's *d *= 0.77 (95% CI: 0.19-1.34). Similar effects were noted on measures of quality of life and IBS-related fear and avoidance behaviors. Improvements in the ICBT group were maintained at 12-month follow-up. The ICBT condition was found to be more cost-effective than the waiting list, with an 87% chance of leading to reduced societal costs combined with clinical effectiveness. The cost-effectiveness was sustained over the 12-month period.

**Conclusions:**

ICBT proved to be a cost-effective treatment when delivered to a sample recruited from a gastroenterological clinic. However, many of the included patients dropped out of the study and the overall treatment effects were smaller than previous studies with referred and self-referred samples. ICBT may therefore be acceptable and effective for only a subset of clinical patients. Study dropout seemed to be associated with severe symptoms and large impairment. Objective and empirically validated criteria to select which patients to offer ICBT should be developed.

**Trial Registration:**

ClinicalTrials.gov: NCT00844961

## Background

Irritable bowel syndrome (IBS) is a functional bowel disorder characterized by recurring symptoms of abdominal pain or discomfort, accompanied by diarrhea or constipation [1]. For a majority of the affected, IBS is chronic and leads to impaired quality of life [2-4]. Compared to normal controls, IBS-patients are about three times more likely to be absent from work [5] and they utilize health care resources at almost double the cost [6]. Given the high prevalence of IBS, ranging between 5 and 11% [7], the societal costs of IBS are substantial [8].

Cognitive behavior therapy (CBT) is considered the most well-studied psychological treatment for IBS [9], but one limitation is that CBT is rarely available in routine care of IBS [10]. Several factors contribute to this, e.g. the lack of trained therapists, high costs of delivering the treatment, and the practical difficulties for patients of scheduling weekly visits at a clinic. To increase the availability of CBT for IBS, our research group has conducted two studies investigating CBT for IBS where participants had therapist contact via the internet (ICBT). In ICBT, patients learn about the treatment interventions by reading self-help texts that contain both educational material and instructions on how to perform the exercises that constitute the treatment. The general principle is that the treatment should reflect face-to-face therapy in terms of content, but using an online therapist to guide the participants through the course of the treatment. The format allows for large patient volumes to be treated and an increasing number of controlled studies indicate that for common psychiatric disorders ICBT is as effective as face-to-face delivered treatment [11-13]. In our trials of ICBT for IBS the treatment was found to be significantly more effective than a waiting list control condition [14] and a treatment based on stress and symptom management [15]. A follow-up study also showed maintenance of improvements over a 15-18 months period [16] and that the treatment was associated with considerable long-term societal cost-savings [17]. The treatment, which is based on exposure to IBS symptoms and mindfulness exercises, has also been evaluated in an uncontrolled pilot study using a group treatment format, showing similar effects [18].

These results indicate that an effective treatment can now be offered to a large number of IBS patients. However, the generalizability of our studies and other studies investigating CBT for IBS with reduced therapist time [19-22] is limited by the fact that study participants have mostly been recruited by referral and self-referral. It could be assumed that these methods of recruitment result in samples of IBS patients who are more willing and able to participate in a minimal contact therapy and who are also more responsive to CBT in general.

In the present study we offered the ICBT that we have previously evaluated with self-referred IBS patients [14,15] to consecutively recruited patients at a gastroenterological outpatient clinic. Our aim was to investigate the acceptability, effectiveness, and cost-effectiveness of the treatment for a representative sample of tertiary care IBS patients. Our primary hypothesis was that, compared to a waiting list, ICBT would lead to greater reductions in IBS-symptoms at the end of treatment. We also hypothesized that ICBT would be superior to a waiting list in terms of cost-effectiveness, improvement in symptom-related anxiety, quality of life, and general functioning. Additionally, the improvements gained from treatment were hypothesized to be maintained 12 months after treatment.

## Methods

This study is reported in accordance with the CONSORT statement for non-pharmacological trials [23] and was approved by the regional ethics committee in Stockholm, Sweden.

### Sample and recruitment

#### Power considerations

In our previous study of ICBT for IBS using a waiting list control, we had a between-groups effect size (Cohen's *d*) of 1.21 (95% CI: 0.73-1.66) on the primary outcome measure at post-treatment [14]. Based on the lower limit of the confidence interval, we powered this study to have an 85% chance to detect an effect size of at least 0.80, comparing treatment to a waiting list, on the primary outcome. This gave a sample of at least 30 participants per study condition.

#### Inclusion criteria

Participants were eligible for the study if they a) had their first visit at the recruiting clinic and were diagnosed during the recruitment period, b) had IBS symptoms as their primary reason for consultation, c) fulfilled Rome III-criteria for IBS [1], d) were between 18 and 65 years old, e) had no presence of current or previous inflammatory bowel disease, and f) lived in Stockholm County. Eligible patients were excluded if they g) reported debut of IBS symptoms after 50 years of age and were judged to require continued monitoring at the clinic, h) suffered from such severe diarrhea that IBS-symptom modifying drugs with psychotropic effects, such as tricyclic antidepressants or selective serotonin reuptake inhibitors, were judged to be the treatment of choice, i) could not read or write Swedish j) did not have access to the internet, k) were judged to be highly unsuitable for ICBT for somatic or psychological reasons as assessed by the gastroenterologist, or l) were not willing to participate in the study.

#### Recruitment procedure

All patients were consecutively recruited at a single gastroenterological clinic located in Stockholm, Sweden. Patients came to the clinic by referral or by self-referral. All patients who had their first visit at the clinic between November 19, 2008, and May 13, 2009 were eligible for the study. The recruitment ended in June 22, 2009, so the patients had to have been diagnosed before that date to be included in the study. As our aim was to recruit patients who attended a regular gastroenterological clinic and minimize selection bias, no information about the study was spread through advertisements or to other caregivers in Stockholm. Four gastroenterologists with 10 to 30 years of specialist experience were responsible for the recruitment. After diagnosing a patient with IBS and confirming eligibility criteria the gastroenterologist informed patients about the study. Patients who were willing to participate signed a written consent form. Some patients were contacted by telephone and offered to participate in the study. Reasons for telephone contact could be that the patient did not return for a second visit to receive their diagnosis, that he or she could not immediately decide whether to participate in the study, or that the gastroenterologist had omitted to inform the patient about the study during the visit at the clinic. Patients that agreed to participate in the study via telephone signed the written consent form at the ensuing psychiatric assessment (see below). All included patients were given standardized information about IBS and basic dietary and lifestyle advice on how to manage their IBS (i.e., treatment as usual). If appropriate they were also prescribed medication and/or given information about over-the-counter drugs. To ensure that this basic IBS management would have had its effect before patients begun their participation in the study, the pre-treatment assessment was conducted at least one month after inclusion.

**Figure 1:**
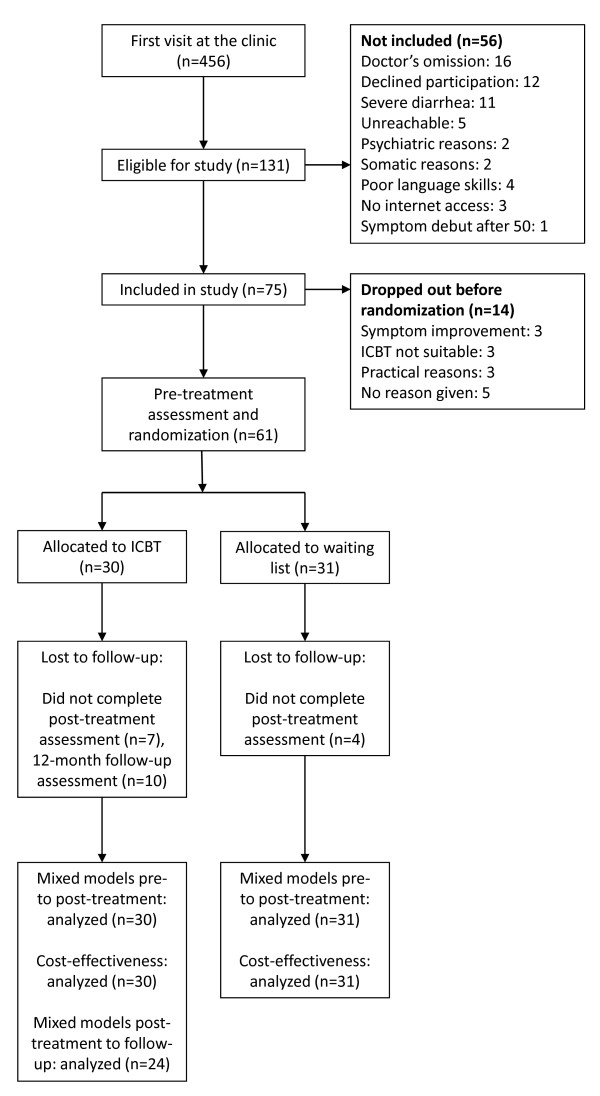
**Participant flow through the trial**.

After the recruitment period had ended, the study's coordinating gastroenterologist (P.L.) reviewed all visits at the clinic during the recruitment period to check if all eligible patients had been considered for the study. The clinic's computerized medical record system allowed us to filter out all patients who did not fulfill eligibility criteria. A total of 456 patients had their first visit at the clinic during the recruitment period, and 131 of these were eligible according to their medical record. However, for 16 eligible patients there were no indications that they had been considered for the study and it could not be determined why they had not been included. Upon reviewing the records of these 16 patients, no obvious biases were found that could explain these breaches of the study protocol. Of the 115 patients that were considered for the study, 35 were excluded based on criteria g-j (reasons for exclusion are detailed in Figure 1), 5 patients could not be reached by telephone within the recruitment period, and 75 were included in the study. Of the 75 patients that were included at the clinic, 14 withdrew in the period between inclusion and pre-treatment assessment (reasons for withdrawal are given in Figure 1) and 61 were randomized. Demographics for the randomized participants are given in Table 1 and participant flow through the study is detailed in Figure 1.

**Table 1:** Sample characteristics

	Total sample (n = 61)	Treatment group (n = 30)	Waiting list (n = 31)
% Female	74%	77%	71%
Age (SD)	34.9 (11.3)	33.5 (11.2)	36.3 (11.3)
Years with IBS (SD)	11.5 (11.8)	11.7 (12.7)	11.3 (11.1)
IBS subtype %			
Constipation	21%	20%	23%
Diarrhea	30%	27%	32%
Mixed	49%	53%	45%
Married %	81%	77%	84%
Education %			
Elementary school	5%	7%	3%
High school	30%	33%	26%
University degree	62%	60%	65%
Doctoral degree	3%	0%	7%
Employment status %			
Employed	79%	73%	84%
Unemployed	3%	7%	0%
Student	15%	17%	13%
Retired	3%	3%	3%

### Randomization

Randomization was performed by sending a list of anonymous participant identification numbers to one of the co-authors (E.H.) who had no involvement in the recruitment, administration, assessment, or treatment of the patients. A true random number service (http://www.random.org) was used to allocate the participants to either ICBT or waiting list, using simple randomization with no restrictions. After randomization the anonymous participant identification numbers were returned together with their allocation to the first author who coordinated the study (B.L.). Since all outcomes that were waiting list controlled were self-assessed, there was no concealment of allocation.

### Treatment condition

The ICBT in this study is based on a treatment protocol that has been evaluated previously in group format and delivered via internet [14,15,18]. The treatment aims to break the vicious cycle between avoidance behaviors, symptom severity, and functional impairment. It is based on the proposed central role of symptom-related fear and associated avoidance behaviors in IBS [24]. Since fear alters motility [25] and directs attention towards threat [26], the association between symptom-related stimuli and fear constitutes a solid foundation for positive feedback loops between stress and IBS symptoms, as well as an increased awareness of symptoms. Use of avoidance behaviors to cope with this fear may also lead to long-term aggravation of symptoms because of increased general stress and strengthened negative valence of symptoms. Several studies have confirmed the impact of symptom-related fear and avoidance behaviors on symptom severity and quality of life in IBS [24,27-30].

In comparison to other CBT-protocols for IBS, the main unique feature of the CBT in this study is its strict reliance on acceptance of IBS symptoms and related cognitions and feelings through exposure exercises combined with mindful awareness. It is inspired by the "third wave" of CBT that includes treatments such as acceptance and commitment therapy (ACT) and dialectic behavior therapy (DBT)[31]. In this treatment, exposure is proposed to serve two purposes. Similarly to how exposure has been used in other studies of CBT for IBS [21,32,33], it will result in long-term extinction of the fear response to IBS-related stimuli, leading to reduction in symptoms. But in accordance with reasoning within ACT [31,34], exposure also serves to increase behavioral flexibility in the presence of IBS-related stimuli, while being aware of and accepting the feelings elicited by the stimuli. By practicing a behavioral repertoire that is not influenced by fluctuations in IBS symptoms and mood, patients will perceive symptoms as less threatening to their ability to function.

The treatment also includes mindfulness training. Mindfulness-based stress reduction for IBS has been evaluated in two recent studies [35,36] with mixed results. While patients in both studies experienced improvement in quality of life only one of the studies demonstrated effects on IBS symptoms [36]. Mindfulness training in this treatment does not serve to decrease stress but rather to practice acceptance of aversive inner experiences while engaging in flexible and more functional behavioral responses to these experiences, similarly to how it is used in ACT and DBT [37,38].

The treatment in this study lasted for 10 weeks and was divided into five successive steps. The content of each step is presented in Table 2. Patients had to report that they had worked through a treatment step to get access to the next. Patients were encouraged to work through steps 1-4 during the first half of the treatment and to spend the latter half of the treatment on step 5, in which exposure exercises were introduced. The number of patients in the treatment group reaching each step is presented in Table 2. During treatment, patients also had access to an online closed discussion forum where they could discuss their treatment with each other.

**Table 2:** Description of the treatment steps and number of patients reaching each step

Step	Contents	# of patients
1	Introduction to the treatment and two mindfulness exercises. 1. A 15-minute exercises during which the patient observes and labels inner and outer experiences (practiced once daily). 2. A 20 second exercise that brings the patient into immediate awareness of current thoughts, GI symptoms, feelings, and behavioral impulses (practiced several times daily).	7(23%)
2	Explanation of a psychological model of IBS. The learning of symptom-related fear through negative experiences of symptoms. The effect of anxiety on gastrointestinal functioning and how it increases awareness of threatening stimuli - specifically IBS-related stimuli.	6 (7%)
3	The role of negative thoughts in exacerbating IBS-related fear. A mindful and accepting stance towards negative thoughts and experiences is proposed as an alternative to attempts to control these experiences.	3 (10%)
4	Explanation of how IBS-related avoidance and control behaviors maintain the fear of awareness of IBS-symptoms. Patients record their own IBS-related behaviors.	5 (17%)
5	Behavior change and exposure, chiefly divided into three categories. 1) Reduction or removal of behaviors that serve to control symptoms, such as repeated toilet visits, distraction, eating certain foods, resting, and taking unprescribed medications. 2) Exposure to symptoms by engaging in activities that provoke symptoms, such as eating certain foods, physical activity, and stressful situations. 3) Exposure to situations where symptoms are unwanted, such as attending a meeting when experiencing abdominal pain or riding the bus with fear of losing control of the bowels. Instructions on how to use mindfulness during exposure. By observing and labeling their environment during exposure, i.e., aversive, neutral, and positive internal and external stimuli, patients will counter distraction from and suppression of thoughts and emotions. By attending to any impulses to flee the situation or decrease the intensity of symptoms they will also be less inclined to act on these impulses. In the last week of treatment all patients got access to a text that discussed how to handle relapses into avoidance behaviors and how to maintain a widened behavioral repertoire.	13 (43%)

Three clinical psychologists managed the online therapeutic contact in this study. Therapist contact was usually initiated by the participants who were encouraged to send at least one message per week about their work with the treatment to their therapist. Patients were given feedback within 1-2 days after they had posted a message. On average, patients sent 8.2 (SD = 4.7, range 0-18) messages to their therapist and received 11.8 (SD = 5.7, range 1-21) messages from their therapist. The therapists spent a weekly mean of 7.3 minutes (SD = 5.2, range 0-24.5) per patient. Three types of feedback were generally given by the online therapists: 1) Corrective psychoeducational information if the patient's answers indicated that he/she had not fully grasped the rationale for the treatment. 2) General support to maintain or increase the intensity of the patient's work with the treatment. 3) Guidance on how to perform and evaluate the exercises prescribed by the treatment.

### Waiting list condition

Patients randomized to waiting list were crossed over to treatment after the post-treatment assessment was concluded. During the waiting list period these patients were also offered the opportunity to communicate with each other using an online closed discussion forum. The discussion forum was included as a basic control for the effects of weekly activity and attention. Online discussion forums have been shown to alleviate distress associated with breast cancer [39]. However, there was almost no discussion forum activity in the waiting list condition.

### Data collection

All measures were administered online, a method which has been shown to be reliable and as valid as traditional paper-and-pencil administration [40,41]. The outcome measures were assessed at three time points; pre-treatment, post-treatment, and 12-month follow-up (ICBT only). Additional data that are not part of this report were also collected. Waiting list group participants completed assessments immediately after and 12 months after having been crossed over to and finishing treatment. Psychiatric assessments of all patients were also conducted before randomization and at 12-month follow-up. This psychiatric assessment, which was performed during a visit at a psychiatric clinic in Stockholm, was not part of the inclusion process and did not guide the treatment intervention.

### Primary outcome

The primary outcome measure of the study was the Gastrointestinal Symptom Rating Scale-IBS version (GSRS-IBS) [42]. The GSRS-IBS is a version of the Gastrointestinal Symptom Rating Scale [43] extended with questions about Rome I-criteria for IBS. The GSRS-IBS comprises 13 items covering severity of gastrointestinal symptoms, including pain, bloating, diarrhea, constipation, and satiety. Each item is scored between 1 (no discomfort at all) and 7 (very severe discomfort), rendering a total score between 13 and 91. The GSRS-IBS has demonstrated adequate psychometric properties for the different symptoms that are assessed, with an internal consistency (Chronbach's *α*) ranging between .74 and .85 [42]. Since IBS symptoms are known to vary considerably over time [44], the mean score of four weekly assessments of GSRS-IBS was used to get reliable estimates of the participants' symptom levels at each assessment point.

### Secondary outcomes

Health economic data were collected using the Trimbos and Institute of Medical Technology Assessment Cost Questionnaire for Psychiatry (TIC-P) [45]. This questionnaire assesses monthly health care consumption (direct medical costs) and time spent in other health promoting activities (direct non-medical costs). The TIC-P also assesses monthly sick-leave, reduced work capacity at work and in the domestic realm, and employment status (indirect non-medical costs).

The Irritable Bowel Syndrome Quality of Life Instrument (IBS-QOL) [46] was used to assess the impact on quality of life for patients with IBS, and includes domains such as dysphoric thoughts, symptoms interference with activity, food avoidance, and impact on relationships. The IBS-QOL consists of 34 items scored between 1 and 6 and the total score is transformed to a 0 to 100 scale, where 0 represents minimum quality of life and 100 represents maximum quality of life. The IBS-QOL shows high internal consistency (Chronbach's *α *= .95) and test-retest reliability (*r *= .86) [46].

The Visceral Sensitivity Index (VSI) [28] measures the cognitive, affective, attentional, and behavioral dimensions relating to fear of IBS symptoms and associated situations. The VSI comprises 15 items that are scored between 0 and 6, with a total score between 0 (minimum GSA) and 75 (maximum GSA). The VSI has demonstrated high internal consistency (Chronbach's *α *= .90 - .92) [28] and most notably it has been shown to be a key explanatory variable in predicting IBS diagnostic status [24].

The Sheehan Disability Scales [47] assess symptom induced disability in three domains: social, work, and family from 0 (no disability) to 10 (severe disability), with a total score between 0 and 30. The Sheehan Disability Scales show high internal consistency with Chronbach's *α *= 0.89 [48].

At 12-month follow-up patients were also asked what type of health care, if any, they had utilized because of IBS-symptoms since the treatment had ended.

### Analysis

Main outcome continuous variables were analyzed in SPSS 19 using a linear mixed effects model fitted with full information maximum likelihood estimation (FIML) [49]. Linear mixed models take into account the non-independence of repeated-measures data and individual heterogeneity by including random effects in the model. The superior qualities regarding missing data as well as increased power compared to the traditional repeated measures ANOVA make mixed models the preferred choice for longitudinal data analysis [50]. Linear mixed models were used to examine the difference in rates of change between the ICBT and waiting list from pre- to post-treatment as the fixed effects interaction between group and time. Model selection was determined analytically by means of likelihood ratio tests and included random intercepts. Error terms were held equal across time and were not correlated. Mixed models were also used to examine the effect of time between post-treatment and 12-month follow-up for the ICBT group. Sensitivity analyses were conducted by including treatment seeking within the follow-up period as a covariate in the analyses. These models used the same covariance structures as above.

All available data from all participants and measurement points were used, which made this an intent-to-treat analysis. Under the assumption of data missing at random (MAR), FIML provides unbiased parameter estimates compared to traditional methods (such as last observation carried forward) even with a substantial proportion of missing data [51]. FIML under the assumption of MAR requires that observed variables that are associated with the likelihood of data missingness are included in the analysis [52]. Prior to conducting the primary analysis, the missing data mechanism was assessed by exploring the relationships between baseline characteristics and the presence of missing data in the sample.

All confidence intervals are given with a 95% margin. Between-groups effect sizes were calculated as standardized mean differences (Cohen's *d*). Conservative estimates of effect sizes were obtained by replacing missing post-treatment data with pre-treatment data.

### Cost-effectiveness analysis

The same methodological approach in the cost-effectiveness analysis as in our previous study [17] was used to make the results comparable across studies. Self-reports from TIC-P were used to estimate the costs generated by the participants. Medication costs were based on the market price in Sweden. Costs of health care visits were estimated using national tariffs in Sweden. Productivity losses were based on the human capital approach, which means that the value of reduced work productivity were calculated using average gross earning [53]. Gross earnings were estimated using the average salary in Sweden by education level. Domestic losses were estimated at $ 13.17 in accordance with our previous study [17] and originally estimated by Smit et al. [54]. Costs were converted to US$ using purchasing power parities for the reference year 2010 [55]. The statistical cost data analyses were conducted using STATA 11/IC and SPSS 19 in three steps:

First, cost means between treatment and control group at pre-treatment, post-treatment and at 12-month follow-up were estimated. Similar to our previous study [17], missing cost data was imputed in a last-observation-carried-forward manner, and all costs were extrapolated to a 12-month period. ANCOVA with post-treatment costs as dependent variables and pre-treatment costs as covariates were conducted to assess between-group differences in change from pre- to post-treatment. Paired t-tests were used to test within-group differences from pre- to post-treatment. Bootstrap replications (5000 replications) were used as we expected the cost-data to be non-normally distributed [56].

**Figure 2:**
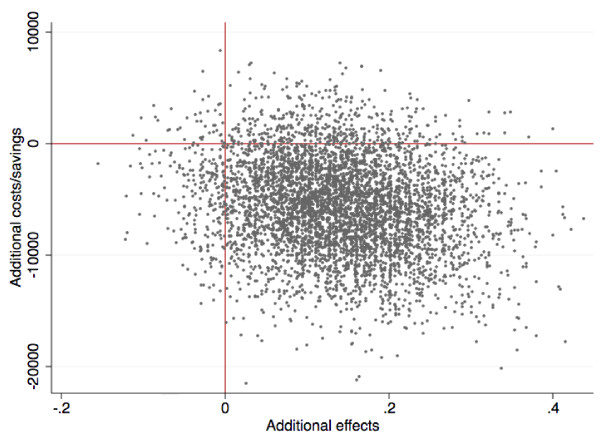
**Cost-effectiveness plane**.

Second, incremental cost-effectiveness ratio (ICER) was estimated computing differences in costs and effects between both conditions: (C(ICBT) - C(WL))/(E(ICBT) - E(WL)). C refers to the pre-post cost differences and E is the proportion of patients showing clinically significant improvement in both conditions [53]. Similarly to our previous cost-effectiveness study, clinically significant improvement was defined as at least 50% symptom reduction on the GSRS-IBS. This calculation was repeated 5000 times (for each bootstrap sample) generating a scatter of simulated ICERs, which represents the probability of the treatment being cost-effective. Figure 2 presents a scatterplot of the simulated ICERs. If a majority of the ICERs appear in the southeast quadrant of the figure, lower societal costs are achieved alongside beneficial health gains. From a cost-effectiveness perspective, this is the most favorable outcome. ICERs located in the northeast quadrant indicate that better health gains are achieved with ICBT but to higher societal costs compared to no treatment. ICERs in the northwest quadrant indicate that ICBT is inferior to no treatment and results in higher societal costs. ICERs located in the southwest quadrant indicate that ICBT is associated with inferior health gains but produces lowered societal costs compared to no treatment.

Third, the economic long-term impact using 12-month follow-up data was investigated. Since there was no experimental control at 12-month follow-up, the treatment group's follow-up data was imputed and compared with the post-treatment data of the control group. This was done to test whether the extrapolation to a 12-month period was a reliable analysis procedure.

### Exploratory analysis

Clinical guidelines recommend that IBS patients should be referred to psychological treatments if they show anxiety, activity disruption or poor coping strategies [7,57]. Since patients were recruited without taking these factors into consideration, we tested whether low levels of anxiety and poor coping strategies (as measured by the VSI) or activity disruption (as measured by the IBS-QOL and Sheehan Disability Scales) predicted low treatment response (as measured by change score on the GSRS-IBS from pre- to post-treatment). This was done in two ways. First, median split was used to compare the outcome between patients with scores above the median and patients below the median on each of the predictor variables, using t-tests. Second, each of the predictor variables was correlated with the outcome, while controlling for the pre-treatment value of GSRS-IBS. These analyses were only performed on the ICBT patients who participated in the post-treatment assessment.

## Results

### Missing data

A total of 50 of the 61 randomized patients (82%) completed the post-treatment assessment, 23 of 30 (77%) in the ICBT group and 27 of 31 (87%) in the waiting list. Statistical tests of baseline characteristics showed that patients with missing data tended to score higher on GSRS-IBS (*m *= 48.1, *SD *= 12.5 vs. *m *= 40.9, *SD *= 11.2; *t*_60 _= 1.90, *p *= .06) than patients that completed the post-treatment assessment. Within the ICBT condition, patients that dropped out scored lower on the IBS-QOL at pre-treatment (*m *= 52.2, *SD *= 26.1 vs. *m *= 72.0, *SD *= 17.1; *t*_28 _= 2.36, *p *= .03). The pre-treatment scores of GSRS-IBS and IBS-QOL were therefore included as candidate covariates in the analyses. If these variables significantly improved the model they were retained in the final model. Of the 30 patients in the ICBT group, 20 (67%) completed the 12-month follow-up assessment. One of these patients did not complete the post-treatment assessment, but was included in the analysis of change between post-treatment and follow-up resulting in 24 patients in this analysis. Of these 24 patients 5 had missing data at either post-treatment or 12-month follow-up. Because of the small groups, no analyses were conducted to examine baseline differences between the 5 patients with missing data and the 19 with complete data.

### Group comparisons

Results on the outcome measures at pre- and post-treatment for both groups are displayed in Table 3 together with coefficients for the fixed effects of group, time, interaction effect of group and time, and retained pre-treatment covariates. The mixed effect models demonstrated significant interaction effects between group and time on all outcome measures. The between groups effect sizes at post-treatment were in the upper medium range (*d *= 0.73-0.79) for all measures except the Sheehan Disability Scales (*d *= 0.19). Replacing missing post-treatment data with pre-treatment data yielded considerably lower effect sizes on the GSRS-IBS (*d *= 0.23), IBS-QOL (*d *= 0.26), and VSI (*d *= 0.26) and a negligible between-groups effect on Sheehan Disability Scales (*d *= -0.05).

**Table 3:** Continuous outcome measures

	Treatment			Waiting list			Effect size	Mixed models		
	
	*n*	*m*	*SD*	*n*	*m*	*SD*	Cohen's *d *(95% CI)	Effect	*B*	*p*
**GSRS-IBS**										
Pre	30	44.6	11.1	31	39.8	12.0		Group	4.9	.12
Post	23	31.0	10.2	27	40.9	14.5	0.77 (0.19 - 1.34)	Time	0.6	.63
FU	19	29.9	12.6					G×T	-11.7	.001
										
**IBS-QOL**										
Pre	30	67.4	20.9	31	76.1	18.8		Group	-8.8	.09
Post	23	82.6	13.4	27	67.4	23.1	0.79 (0.20-1.35)	Time	-7.2	.001
FU	20	87.6	11.8					G×T	16.6	.001
										
**VSI**										
Pre	30	32.5	18.0	31	27.5	16.3		Group	5.0	.22
Post	23	14.1	15.1	27	26.2	17.9	0.73 (0.14 - 1.29)	Time	-11.7	.01
FU	20	15.5	15.9					G×T	-14.6	.001
								COV	0.3	.03
										
**Sheehan** **Disability Scales**										
Pre	30	11.9	8.1	31	8.7	6.3		Group	3.2	.08
Post	23	6.4	6.7	27	7.8	7.6	0.19 (-0.37 - 0.75)	Time	-4.4	.01
FU	20	5.2	6.6					G×T	-4.2	.002
								COV	0.1	.04

Unstandardized (*B*) coefficients and their *p*-values are given for fixed in effects in linear mixed models using all data from pre-treatment and post-treatment and all individuals (*n *= 61). The coefficient associated with the main effect of group denotes average score difference between the groups at the pre-treatment assessment. The coefficient associated with the effect of time denotes the average change score from pre to post-treatment across both groups. The coefficient associated with the effect of G × T (group × time) denotes the difference between groups in change score from pre- to post-treatment. COV is the effect of the pre-treatment value of GSRS-IBS, as a retained covariate in the model, on the post-treatment value. Group_0 _= Treatment Group, Group_1 _= Waiting List, Time_0 _= Pre-treatment, and Time_1 _= Post-treatment. Effect sizes are calculated as standardized mean difference (Cohen's *d*) at post-treatment. *GSRS-IBS*: Gastrointestinal symptom rating scale-IBS version. *IBS-QOL*: The Irritable Bowel Syndrome Quality of Life Instrument (IBS-QOL). *VSI*: The Visceral Sensitivity Index.

### Follow-up

Table 3 displays the 12-month follow-up scores for the ICBT group. The mixed effects models gave only one significant effect of time, on the IBS-QOL (*B *= 4.5, *t*_20.1 _= 2.2, *p *= .04). This positive coefficient indicates further improvement on the IBQ-QOL for the ICBT group during the follow-up period while scores on the other outcome measures were maintained. There were no significant effects of time on the GSRS-IBS (*B *= -2.4, *t*_18.7 _= -1.1, *p *= .27), VSI (*B *= -0.4, *t*_19.5 _= -0.26, *p *= .80), or the Sheehan Disability Scales (*B *= -1.4, *t*_21.6 _= -1.2, *p *= .25).

Seven of the 20 patients (35%) reported further treatment seeking because of IBS during the follow-up period, 6 had consulted a physician and 1 had sought complementary treatment, none had sought further psychological treatment. Sensitivity analysis did not indicate any effect of further treatment seeking on maintenance of improvement during the follow-up period.

### Cost-effectiveness

Annual cost means are presented in Table 4. We found no significant between-group interaction effects for any cost domain, but a trend regarding work-cutback (*F *= 3.17, *p *= .08) favoring the ICBT condition was observed. Paired t-tests showed a within-group cost reduction in domestic loss (*p *= .01) and reduced medication costs (*p *= .05) for the ICBT condition. The control group made a significant increase in costs related to work-cutback (*p *= .03). There were no within-group differences for the treatment group between post-treatment and the 12-month follow-up.

**Table 4:** Mean annual costs

	Pre-treament				Post-treatment				Follow-up	
	
	Treatment		Waiting list		Treatment		Waiting list		Treatment	
	***m***	***(se)***	***m***	***(se)***	***m***	***(se)***	***m***	***(se)***	***m***	***(se)***

Direct medical costs	2487	(633)	2115	(689)	1148	(301)	1453	(417)	1868	(566)
Health care visits	2455	(634)	2082	(687)	1405	(374)	1412	(413)	2284	(706)
Medications	33	(8)	33	(11)	21	(8)	42	(18)	37	(17)
										
Direct non-medical costs	504	(265)	88	(42)	150	(105)	338	(243)	405	(177)
										
Indirect non-medical costs	14817	(3554)	13440	(2852)	12729	(3103)	16233	(3124)	12437	(3134)
Unemployment	10204	(3435)	7054	(2937)	8747	(3248)	8465	(3155)	8747	(3248)
Sick-leave	3492	(1531)	5547	(1439)	3028	(940)	6295	(1698)	2816	(900)
Work cutback	376	(138)	211	(78)	515	(233)	998	(287)	426	(167)
Domestic	744	(182)	528	(157)	440	(152)	475	(114)	448	(140)
										
Total (excl. intervention costs)	17808	(3866)	15542	(3029)	14306	(3131)	18025	(3367)	15163	(2368)
Intervention costs					709	(78)	298	(26)	709	(78)
										
Total (incl. intervention costs)	17808	(3866)	15542	(3029)	15014	(3112)	18323	(3370)	15871	(3343)

The cost change for the treatment group was $17,808 - $15,014 = -$2,794 and the cost change in the control group was $15,542 - $18,323 = $2,781. Clinically significant improvement was demonstrated by 6 of 30 (20%) patients in the treatment group and 2 of 31 (6%) patients in the waiting list. The incremental cost effectiveness ratio (ICER) was therefore (-2,794 - 2,781)/(0,20 - 0,06) = -39,821. This means that each significant clinical improvement in IBS was associated with a societal cost-reduction of $39,821. The cost-effectiveness plane (Figure 2) gives a more detailed view of the data: 87% of the ICERs were located in the southeast corner, indicating that ICBT was associated with societal cost reductions and also produced health gains compared to the waiting list. There was an 8% probability of the treatment producing superior health gains but to higher societal costs (northeast quadrant). Only 4% and 1% of the ICERs were located in the least favorable quadrants, the southwest and northwest, respectively.

We repeated the analyses and imputed 12-month follow-up data for the treatment group and compared it with the waiting list's post data. A majority (79%) of the ICERs remained in the southeast corner, indicating robustness of the results.

### Prediction of outcome

The VSI, IBS-QOL and Sheehan Disability Scales were used to test if low values predicted low improvement on the GSRS-IBS. Using median split the difference in outcome between high and low scorers on the IBS-QOL, VSI, and SDS were not significant (*p *= .11 - .23). When the predictors were correlated with the symptom improvement score, controlling for GSRS-IBS baseline score, none significantly predicted the change in GSRS-IBS (*p *= .12 - .50, *r *= .15 - .34).

## Discussion

The aim of this study was to evaluate the acceptability, effectiveness, and cost-effectiveness of a treatment previously demonstrated to be efficacious in treating a self-selected sample of IBS patients [14,15]. Patients in this study were consecutively recruited from a gastroenterological clinic and were randomized to ICBT or a waiting list. There was considerable dropout in the study, with only 77% of the ICBT patients participating in the post-treatment assessment and 43% completing the treatment. Compared to the waiting list, the ICBT participants experienced reductions in IBS symptoms, anxiety related to IBS symptoms, as well as improvements in IBS-related quality of life. The effects were more pronounced on the IBS-specific measures than the general measure of daily functioning, probably owing to low pre-treatment values on this measure. Despite the large dropout from the ICBT condition, the treatment produced societal cost-savings compared to the waiting list. Additionally, the cost-savings offset the treatment costs both in short- and long-term.

In our previous trial where we used a waiting list as comparison group, the between-groups effect on GSRS-IBS (*d *= 1.21) [14] was larger than the between-groups effect observed in this trial (*d *= 0.77). Using conservative estimates, by replacing the missing data with pre-treatment assessments, the between-group effect sizes were even lower. Because of the large proportion of missing data and the fact that patients that dropped out were more impaired than the patients that stayed in the trial, the conservative effect sizes are highly influenced by the dropouts. Still, they indicate that for the whole randomized sample, ICBT was clearly less effective than in our previous studies.

The acceptability of ICBT, as measured by the proportions of patients in the ICBT group that stayed in the study (77%) and completed treatment (43%), also differed between this study and our previous studies. In these, 90%-99% of participants completed the post-treatment assessment [14,15] and 69% of the participants completed all five treatment steps [14]. In addition, while 14 of 75 included patients (19%) withdrew from this study before randomization, 0%-3% withdrew from our previous studies after inclusion [14,15].

These differences in effectiveness and acceptability are likely linked to the differences in recruitment method. In our study of group CBT for IBS [18], gastroenterologists at several clinics referred patients that they judged would benefit from treatment and in our previous studies of ICBT [14,15], patients were self-referred. It would be reasonable to assume that the patients in this study, who were offered to participate simply because they visited a gastroenterological clinic, would be less motivated to engage in treatment. Exposure treatment demands a lot from patients and ICBT also requires them to work independently with planning and conducting the exposure exercises. This may have been too challenging for some patients who might not have applied for the study if we would have used self-referral as recruitment method.

According to clinical guidelines, IBS patients with anxiety, poor coping strategies, or functional impairment should be offered psychological treatment [7,57]. Indeed, a recent study showed that improvement after CBT for IBS was mediated through change in avoidance behaviors and negative thoughts about symptoms [58] and that less adaptive IBS behaviors predicted good outcome [59]. However, in this study we did not observe this association. It seems that patients who displayed comparably low levels of avoidance still experienced symptom improvement by changing these behaviors. The study does suggest that large impairment and severe symptoms may be predictive of treatment dropout. However, our previous studies point in another direction. The average pre-treatment scores on GSRS-IBS and IBS-QOL were markedly higher in those studies than in this study, while participants showed larger improvement and treatment adherence [14,15]. This is in accordance with research demonstrating that IBS patients recruited through the internet are more impaired than clinical patients [60]. Thus, it seems that the method of recruitment is more predictive of treatment outcome than symptom severity and level of impairment. More research is needed to establish what minimum level of avoidance behaviors that is required to benefit from a treatment that targets these behaviors. Other types of psychological treatments for IBS, such as hypnosis, self-management, psychodynamic therapy, and cognitive therapy, may target other mechanisms and require other selection criteria.

An important limitation in this study is our use of a waiting list as a comparison group. Although this practice is common in trials of psychological treatments it has been criticized as it does not control for non-specific factors such as expectancy of improvement and attention from a caregiver [61]. Several trials of psychological treatments for IBS have failed to show differential effects on symptoms when using attention control conditions [32,62-64], and a recent study showed that IBS patients even respond to open-label placebo [65]. These studies underline the need for an active control group when evaluating psychological treatments for IBS. However, ICBT did show superiority to an active control in our previous study where 195 self-referred IBS patients were randomized to ICBT or internet-delivered stress management [15].

Another limitation is that although our aim was to evaluate the treatment for a clinical sample, it was not carried out within a clinical context. Patients had to participate in an extensive pre-treatment assessment, including a psychiatric evaluation and four weeks of weekly symptom ratings, and were then randomized to treatment or waiting list. These circumstances are seldom part of clinical care and may have contributed to the high dropout rate. We did not include patients who had severe diarrhea and were therefore judged to require prescription of psychotropic drugs. Eleven of the 131 eligible patients (8%) were excluded from the study for this reason. It would have been preferable to prescribe the drug and after evaluation of its effects possibly offer these patients participation in the study. However, for practical reasons this was not possible, as it would have altered the inclusion procedure and time frame. This further limits the generalizability of the results regarding these patients.

The cost-effectiveness analysis also has some limitations. We did not use the actual salary of the patients but estimated them based on educational levels. This produces errors in the estimated costs but should not affect the ICER as it relies on the randomization. Further, we estimated the cost of treatment based only on the amount of therapist time. This estimation does not include the cost of writing the treatment manual or developing the web platform used for delivering the ICBT, nor does it include the overhead costs of running an ICBT treatment clinic.

## Conclusions

This study shows that ICBT for IBS, based on exposure principles, can be an effective treatment option for some patients at a tertiary care clinic. Patients with large impairment and severe symptoms may be less able or willing to engage in ICBT. From a cost-effectiveness perspective, offering ICBT to clinical patients seems to lead to considerable societal savings compared to no further treatment after regular clinical care, even with large dropout rates and low treatment adherence. For patients that benefit from the treatment, improvements are maintained over a 12-month period. More research is needed to clarify for which patients ICBT based on exposure and mindfulness exercises is best suited.

## Abbreviations

CBT: Cognitive Behavior Therapy; FIML: Full Information Maximum Likelihood; GSA: Gastrointestinal Specific Anxiety; GSRS-IBS: The Gastrointestinal Symptom Rating Scale - IBS version; IBS-QOL: The Irritable Bowel Syndrome Quality of Life Instrument; IBS: Irritable bowel syndrome; ICBT: Internet-delivered Cognitive Behavior Therapy; ICER: Incremental Cost Effectiveness Ratio; MAR: Missing At Random; VSI: The Visceral Sensitivity Index

## Competing interests

The authors declare that they have no competing interests.

## Authors' contributions

BL participated in the design of the study, supervised the treatment of the patients, coordinated the study, and had the main responsibility for writing the treatment manual, conducting data analyses, and drafting of the manuscript. GA participated in the design of the study, analysis of the data, and drafting of the manuscript. EA participated in the design of the study, analyzed the health economic data, and participated in drafting of the manuscript. EH participated in the design of the study, writing the treatment manual, analysis of the data, and drafting of the manuscript. PL supervised the patient recruitment and participated in the design of the study and drafting of the manuscript. SA participated in the design of the study, assessment of the patients, and drafting of the manuscript. CR participated in the design of the study, assessment of the patients, and drafting of the manuscript. NL had main responsibility for designing the study and participated in drafting of the manuscript. All authors read and approved the final manuscript.

## Pre-publication history

The pre-publication history for this paper can be accessed here:

http://www.biomedcentral.com/1471-230X/11/110/prepub
